# Novel citation-based search method for scientific literature: a validation study

**DOI:** 10.1186/s12874-020-0907-5

**Published:** 2020-02-07

**Authors:** A. Cecile J. W. Janssens, Marta Gwinn, J. Elaine Brockman, Kimberley Powell, Michael Goodman

**Affiliations:** 1 Department of Epidemiology, Rollins School of Public Health, Emory University, 1518 Clifton Road NE, Atlanta, GA 30322 USA; 2 Woodruff Health Sciences Center Library, Emory University, Atlanta, GA USA

**Keywords:** Citation, Co-citation, Literature search, Meta-analysis, Systematic review, Keywords

## Abstract

**Background:**

We recently developed CoCites, a citation-based search method that is designed to be more efficient than traditional keyword-based methods. The method begins with identification of one or more highly relevant publications (query articles) and consists of two searches: the co-citation search, which ranks publications on their co-citation frequency with the query articles, and the citation search, which ranks publications on frequency of all citations that cite or are cited by the query articles.

**Methods:**

We aimed to reproduce the literature searches of published systematic reviews and meta-analyses and assess whether CoCites retrieves all eligible articles while screening fewer titles.

**Results:**

A total of 250 reviews were included. CoCites retrieved a median of 75% of the articles that were included in the original reviews. The percentage of retrieved articles was higher (88%) when the query articles were cited more frequently and when they had more overlap in their citations. Applying CoCites to only the highest-cited article yielded similar results. The co-citation and citation searches combined were more efficient when the review authors had screened more than 500 titles, but not when they had screened less.

**Conclusions:**

CoCites is an efficient and accurate method for finding relevant related articles. The method uses the expert knowledge of authors to rank related articles, does not depend on keyword selection and requires no special expertise to build search queries. The method is transparent and reproducible.

## Background

Finding relevant related articles on a specific topic is challenging and time-consuming [1], especially when there is no uniform set of keywords to describe the topic [2]. To quickly find who else has published on the exact topic of a paper, researchers have three options: perform a new literature search, follow the “related articles” link in databases such as PubMed, Web of Science (WOS), or Scopus; or trace the citations to and from the article.

Tracking citations is an intuitive strategy that allows finding articles on the same topic as authors tend to cite papers that are directly related to their work. The reference list of the query article, the so-called “backward citations”, and the newer articles that cite the query article, the “forward citations”, might both include relevant articles [3]. While intuitive, tracking citations is considered inefficient and inaccurate, even as a complement to keyword searching [4, 5]. Tracking citations can only find articles that are connected in a single citation network [6]. A review of 259 meta-analyses, in which researchers aimed to retrieve all published articles on a specific topic, showed that this occurred in less than half (46%) of cases. In 39% of the meta-analyses, the articles were in two disconnected citation networks and in 15% of the meta-analyses in three or more networks [6].

**Fig. 1:**
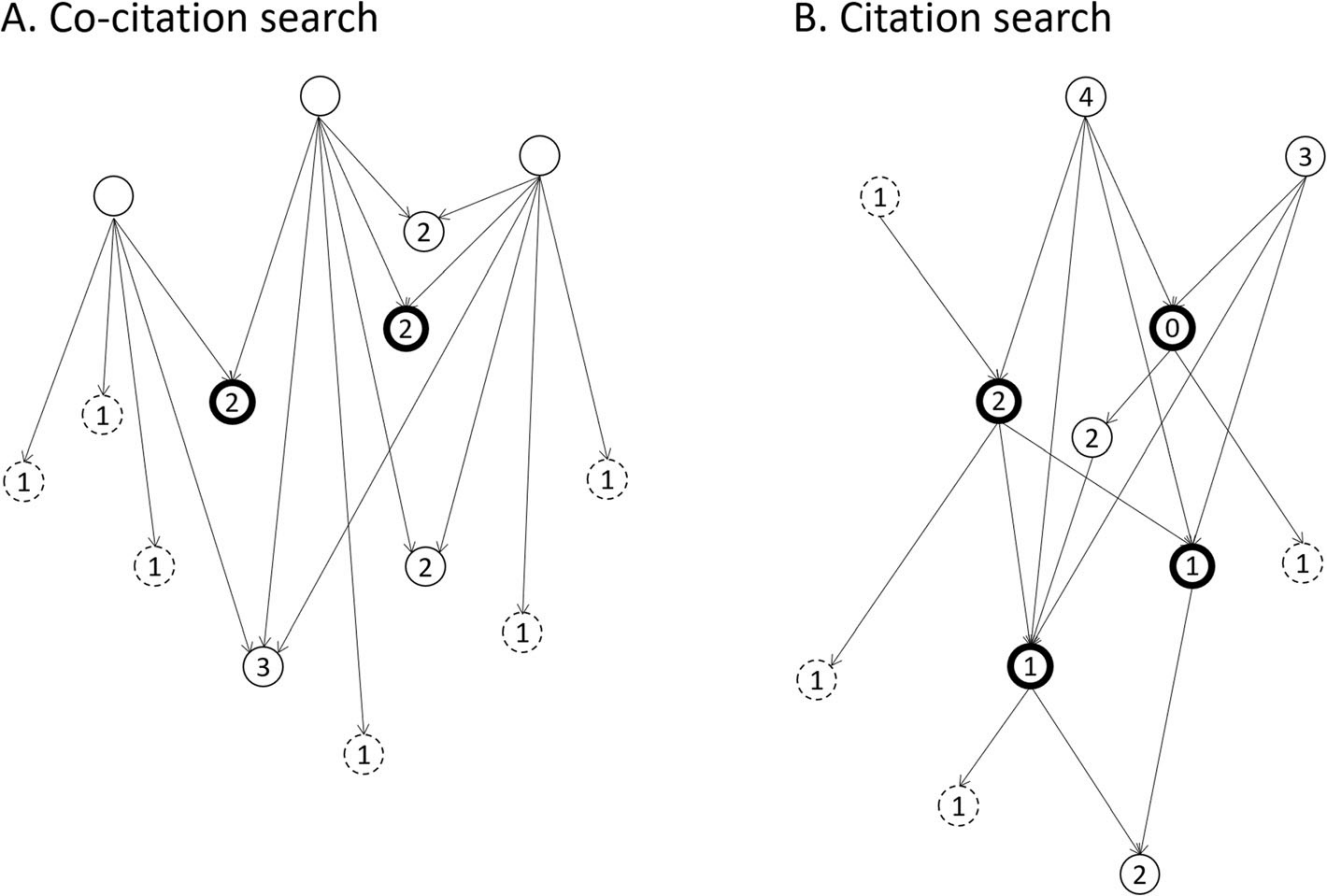
Overview of the search method. Circles represent articles and lines are the citations between them. Arrows indicate the direction of the citation. Bold circles represent the query articles that are used to begin a search. Numbers in the circles indicate the co-citation or citation counts. Dashed circles represent articles that will not be screened for eligibility if the screening threshold is higher than one. Figure is adapted from [7] (distributed under the Creative Commons Attribution 4.0 International License)

We recently developed CoCites, a new search method that finds related articles for one or more articles of interest, termed ‘query articles [7].’ CoCites is based on the principle of co-citation [8] and consists of two searches (Fig. 1). The co-citation search identifies all articles that are cited together with the query article(s) and ranks them in descending order of their co-citation frequency [8]. This search is based on the assumption that articles with a higher co-citation frequency are more likely to address the same specific topic as the query article [8–11]. The citation search finds all articles that cite or are cited by the query articles, and was added to retrieve recently published articles that are not (yet) cited frequently enough to rank higher in the co-citation search.

Co-citation relationships between articles are used in methods that visualize the similarity and clustering among, e.g., articles, authors, and research topics [12–16], and have been previously proposed for literature search methods as well. To find relevant articles for one or more query articles, Belter proposed to screen their citing, cited, co-citing, co-cited articles [17], and to rank these articles based on their number of different relations with the query articles [18]. As each article has a maximum of three relations with a query article (citing/cited, co-citing, co-cited), this ranking method works better with more query articles. Others have examined whether the use of the proximity of (co-)citations within articles efficiently and effectively retrieves relevant articles [19–21]. And again others proposed to search related articles for one or more query articles by screening all citing, cited, co-citing and co-cited articles [22, 23].

In an earlier pilot study, we investigated the performance of CoCites by reproducing the literature searches of published meta-analyses. We found that the method was able to retrieve a median of 82% of the articles included in the meta-analysis. We observed that the percentage of retrieved articles was higher when the two query articles were cited more frequently and when their topics were more similar [7].

In this article, we describe the results of a larger validation study in which we assess both completeness and efficiency of the CoCites searches. As in the pilot study, we tested CoCites’ ability to reproduce the literature searches of published meta-analyses and systematic reviews. We investigated whether the method could retrieve all articles included in the reviews while screening fewer titles. We also assessed citation characteristics that impact the method’s performance.

## Methods

### Detailed description of CoCites

The CoCites method consists of a co-citation and a citation search (Fig. 1). Both searches assume that one or more articles are “known” at the start of the search (Fig. 1, bold circles). These are referred to as “query articles.” When query articles are cited, the reference lists of these citing articles (Fig. 1a, empty circles) contain the articles that are co-cited with the query articles (Fig. 1a, regular and dashed circles). If the two query articles are cited three times in total, there will be three reference lists in which co-cited articles appear 1 to 3 times, as indicated by the numbers in the circles. Users can decide to screen all co-cited articles for their relevance or specify a threshold such as a minimum number of co-citations and ignore e.g., all articles that are co-cited once (Fig. 1a, dashed circles). The citation search finds the articles that are cited in the reference lists of the query articles and those that cite the query articles. When there are four query articles, those articles can cite or be cited by 1 to 4 query articles. The higher the co-citation or citation count, the more likely the article is on a similar topic as the query articles.

### Overview of the study

Additional file 1: Fig. S1 (see Additional file 1) provides an overview of the project, which included several steps. We first obtained a random selection of published systematic reviews and meta-analyses (we refer to both as “reviews”). We then identified the articles that were included in the qualitative or quantitative analysis in each original review (referred to as “included articles”) from which we selected the two mostly highly-cited papers, which were used as query articles. Using a custom-designed web-based tool, we performed the co-citation search and screened the list of publications produced by that search (“screened titles”) to retrieve the articles that were included in the original review (“retrieved included articles” or “retrieved articles”). Retrieved articles that had a co-citation frequency greater than a specified threshold (see analyses) were added to the next query set. We then performed another citation search using the updated query set and screened the new list of titles to retrieve the remaining articles included in the original review. Additional file 1: Fig. S2 (see Additional File 1) illustrates step by step how the web tool works.

### Selection of systematic reviews and meta-analyses

**Fig. 2:**
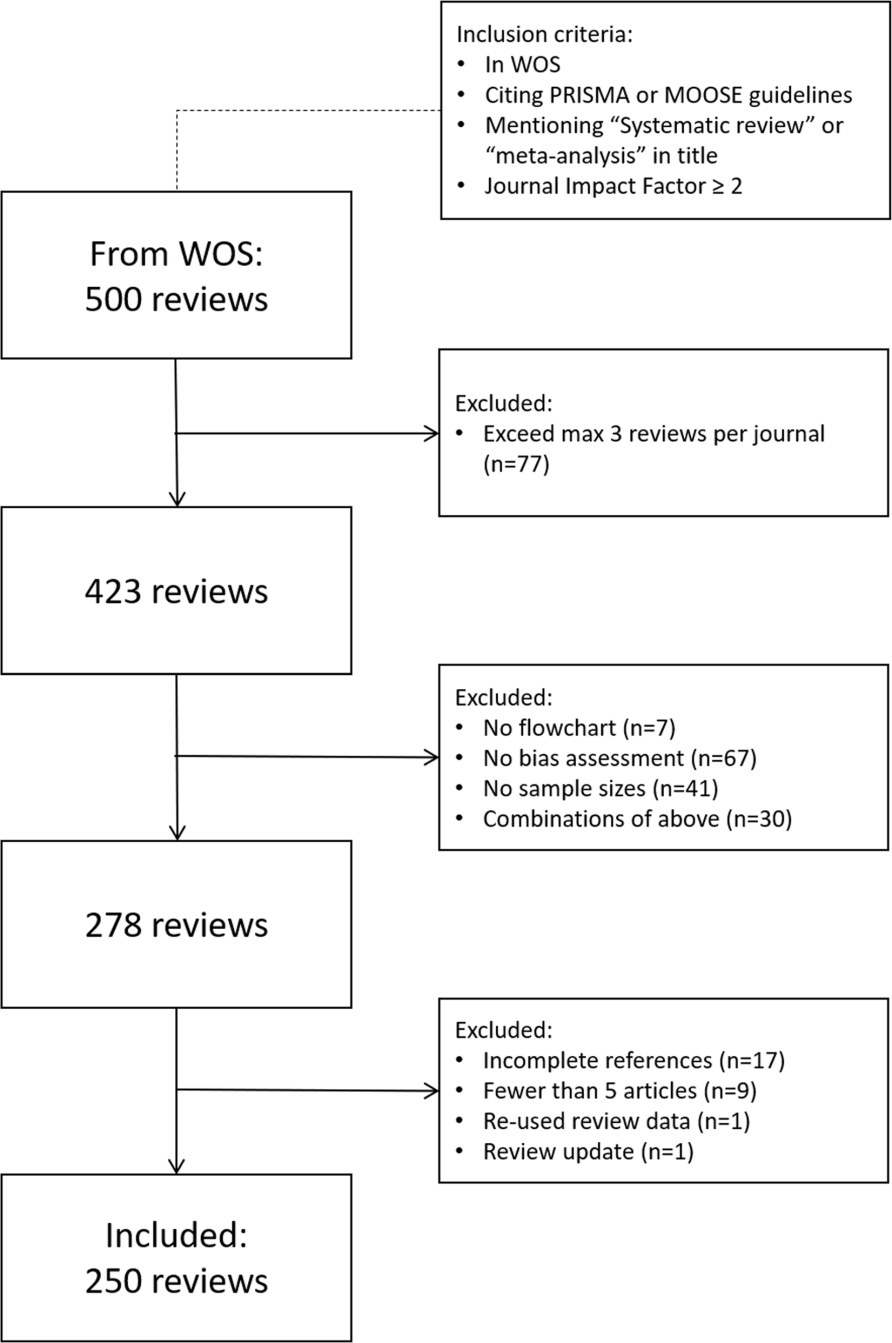
Flowchart for inclusion of systematic reviews. *WOS* Web of Science

Systematic reviews and meta-analyses vary in rigor and quality. They may compare studies that address different research questions (“apples and oranges”), have insufficient search queries, or perform inadequate screening of articles. When evaluating performance of CoCites it is important to focus on the original reviews that meet minimum quality criteria because otherwise it is not clear if the disagreement between the two searches is attributable to the inadequacy of our method or the poor quality of the original review. Therefore, we retrieved systematic reviews and meta-analyses from WOS that cited the PRISMA (Preferred Reporting Items for Systematic reviews and Meta-Analyses) or MOOSE (Meta-analysis Of Observational Studies in Epidemiology) reporting guidelines [24–26], mentioned “systematic review” or “meta-analysis” in the title, and were published in a journal with a 2015 Journal Impact Factor (Journal Citation Reports, Clarivate Analytics) of 2 or higher (Fig. 2). Although the last criterion is arbitrary, it allowed focusing on reviews with higher impact and presumably higher quality. To retrieve a random representative sample of published reviews, we sorted the reviews on their WOS Accession number and selected the top 500 (search date: September 23, 2016).

We noticed that three journals published an exceptionally high number of reviews (Medicine, Scientific Reports, and PLoS One), which led us to limit the number of reviews per journal to a maximum of 3. We only considered reviews that had 1) evaluated the quality of the included articles; 2) reported the numbers of screened and included articles in a flowchart, and 3) reported the sample sizes of all included studies. This information was required for a sub-study investigating the impact of missing data on meta-analyses results. From the reviews that met the above criteria, we further excluded those that had inconsistencies in the references (information in main text not matching reference list), re-used the search results from a literature search that was already in our sample, or included fewer than five articles. All full-text files and supplementary documents were downloaded and stored.

### Retrieval of included articles and selection of highest-cited articles

We downloaded bibliographic data and the reference list for each review. In WOS, the articles in the reference list are stored under a short unique identifier. We extracted the unique identifiers for all references in all reviews, removed duplicates, and downloaded bibliographic data for each article from WOS (date of download: April 25, 2017). In addition to the information on the first author, journal, and publication year, data on each article included the PubMed identification number (PMID) and the number of citations (Times Cited). PMIDs were used as an indicator of whether an article could have been found through a PubMed/Medline search or whether it was likely retrieved through other databases. Missing PMID values were hand-searched in PubMed using several fragments of the titles to verify that PMID values were not available because the articles were not in PubMed or to complete the missing PMID information.

For each review, we documented the end-of-search date and the start date for the search period (if reported), the number of articles screened (after removal of duplicates) and the number of articles included in the qualitative or quantitative analysis in the review. We also identified the included articles in the downloaded reference lists. If the end-of-search date was not reported, we would record the date the review was received, revised, or published instead. If reviews did not report a start search date, we assumed they searched without one.

The two most highly cited articles in each review were identified based on the number of citations at the date the authors had performed their search. We programmed a web-based tool that automatically extracted the citations for each included article in each review and counted the number of citations that were published before the search date reported in the review. The two articles with the highest numbers of citations at the review search date were selected as query articles. When both query articles had more than 1000 citations, we choose the next highest that had fewer than 1000 citations.

### Application of CoCites

The strategy used to develop CoCites’ co-citation and citation searches has been described previously [7] and is diagrammed in Fig. 1. We use a custom-designed, web-based tool to perform the searches automatically (Additional file 1: Fig. S2, Additional file 1), and retrieve data from WOS through its application programming interface (API). For the co-citation search, the tool extracts the reference lists of all unique publications that cite the query articles, counts the number of times each publication appears in all reference lists and ranks them in descending order of co-citation frequency. For the citation search, the program extracts and counts all publications that cite or are cited by the query articles and ranks them in descending order of citation frequency. The removal of duplicates in the co-citation search and the counting of frequencies is based on each article’s unique identifier in the WOS database.

The WOS database includes indexed and non-indexed items. Non-indexed items are those that would not have been included in the database had they not been cited by an indexed article. Examples of non-indexed items include dissertations, reports, and articles in journals that are not covered by WOS. The non-indexed items are available in the WOS database only as cited references and include limited metadata. As their reference lists are not accessible, non-indexed articles are only retrieved when they appear frequently enough in the reference lists of the papers that cite the query articles (co-citation search) or in the reference lists of the query articles themselves (citation search). As all articles included in each review should at least be cited by the review, non-indexed articles are the ones with a missing ‘Times Cited’ count (see below).

### Analyses

We quantified the performance of the search method for four different screening thresholds: (1) articles co-cited at least once (threshold ≥1, i.e., with no exclusions); (2) articles co-cited more than once (threshold > 1); (3) articles co-cited more than once *and* found in more than 1% of the citing publications; and (4) articles that were among the top 100 of all co-cited publications. The choice of these thresholds was based on the pilot study [7], in which the ‘1%’ threshold was investigated to reduce the number of titles needed to screen for highly-cited query articles. For both the co-citation and citation searches, we calculated at each of the four thresholds 1) the percentage of articles in the original review that were retrieved using CoCites and 2) the number of titles that needed to be screened to identify eligible articles. The total number of titles in the search results is the sum of items from the combined co-citation and citation searches; we were unable to reliably remove duplicate records as the database returned the results of the two searches in different formats. When published reviews had used a start search date of 1980 or later, we also excluded earlier publications from our search results for a fair comparison of the number of screened articles.

In our pilot study, we had identified three factors that impacted the performance of CoCites, namely the number of articles that cite the query articles, the percentage of articles included in the reviews that were retrievable through PubMed (had a PMID), and the similarity between query articles. We compared the percentage of retrieved articles between categories of the number of citing articles, percentage of articles in PubMed, and the similarity scores. We quantified the similarity between the query articles using Simpson’s similarity index [27]. This index measures the degree of co-citation between two articles as their number of co-citations divided by the number of citations of the less-cited article. For example, if two query articles are cited 10 and 20 times each, but only three times together, then the similarity score is 3/10 = 0.3. A score of 0.3 means that the two query articles are co-cited in 30% of the citations of the least-cited query article. In our similarity score, the numerator was tied to the search date reported in the review, while the denominator was obtained from the bibliographic download.

Based on these results, we identified a subsample in which we expect the method to retrieve all articles that were retrievable through PubMed. For this subsample, we quantified the percentage of retrieved articles and number of titles in the search results when the two highest-cited or only the highest-cited article was used as query article. At the individual article level, we examined whether articles were more likely to be found when they were older, cited more frequently, having longer reference lists, and when they were indexed in WOS (see above).

Finally, we had assumed that researchers knew the two-highest cited articles when they considered performing their reviews. We explored whether these highest-cited articles could be identified using co-citation searches that started with two query articles that had been cited less frequently. For this analysis, we restricted to the previous subset of reviews and only selected those that included 10 or more articles. From each review, we selected the two articles with the fewest citations but at least ten citations each. We obtained the ranks of the two highest-cited articles and calculated how frequently they and other included articles appeared among the top-ranked results.

## Results

### Description of the reviews

A total of 250 reviews were included, comprising a total of 4761 articles. The authors of the original reviews screened from 18 to 85,714 articles (median 794; Table 1) and included from 5 to 85 articles (median 14). Researchers screened fewer than 200 titles in 17% of reviews, fewer than 500 in 38%, and more than 2000 in 27%.

**Table 1:** Articles screened and retrieved in replicating the results of literature searches in 250 published reviews

	Articles screened, number	Articles retrieved, percentage
In published review	794 (273, 2132)	100
*Co-citation search*		
All co-cited articles	5151 (2709, 10,490)	75.0 (58.2, 87.5)
Co-cited > 1	1119 (544, 2509)	60.0 (45.2, 78.3)
Co-cited > 1%*	696 (461, 978)	56.1 (40.0, 75.0)
100 Top-ranked**	109 (103, 123)	37.5 (22.5, 50.0)
*Citation search*		
Citing or cited by > 1	83 (38, 176)	50.0 (17.9, 75.8)
Total	873 (540, 1204) ***	75.0 (50.0, 90.1)

All values are median and inter-quartile range (IQR). *Co-cited more than once and in more than 1% of the citing articles. The articles retrieved from this search were used to run the citation search. ** Median is higher than 100 because we included all articles that had the same co-citation frequency as the 100th article. *** Sum of results in the co-citation ‘co-cited> 1%’ and citation searches combined, without removing duplicates. See details in methods

### Searching on two highest-cited articles

The query articles had a median of 160 unique citations (inter-quartile range, IQR 80, 262). In 97% of reviews, the query articles were cited more than 20 times and in 68% more than 50 times.

**Fig. 3:**
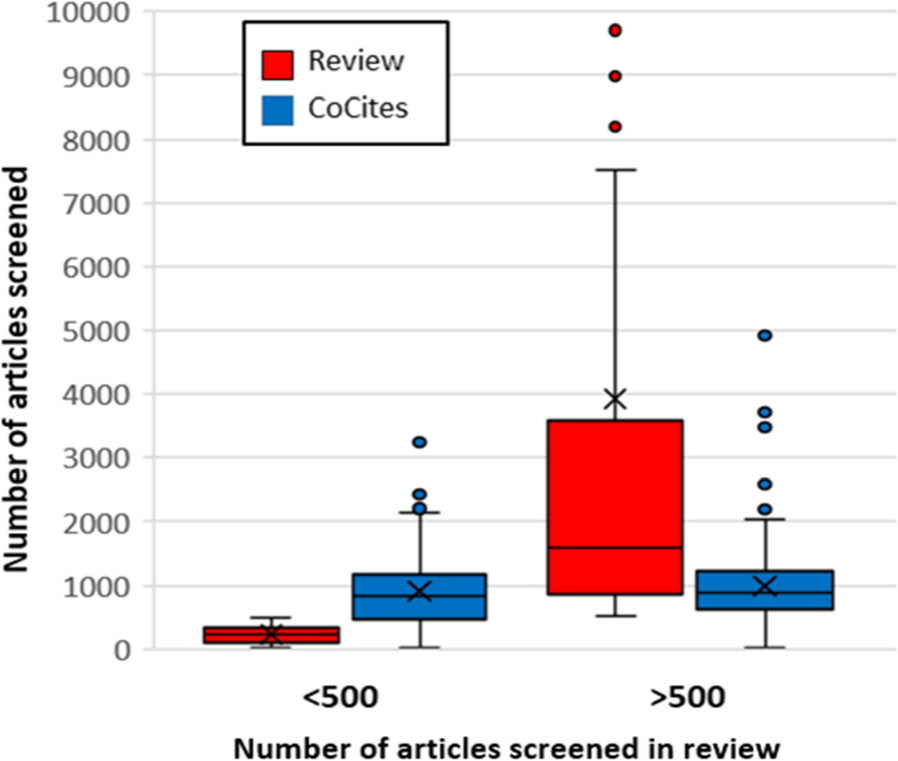
Numbers of articles screened in published reviews versus numbers screened by CoCites’ co-citation and citation searches. Legend: Compared for reviews in which authors screened fewer than 500 articles (left, *n* = 95) and more than 500 articles (right, *n* = 155)

The two highest-cited articles for each review had from 124 to 52,596 co-cited articles (median 5151; Table 1). A median of 1139 articles was co-cited more than once, and in 696 instances they were co-cited more than once *and* in more than in 1% of the citing articles. The co-citation and citation searches combined involved screening a median of 873 articles, which was higher than the median number of articles screened by the authors in their reviews. Figure 3 shows that the two searches combined were less efficient when the review authors had screened fewer than 500 articles, but more efficient when they had screened more.

Co-citation searching retrieved a median of 57% (IQR 40, 75) of the articles at the” 1%” threshold; co-citation and citation searching combined retrieved a median of 75% (IQR 50, 90; Table 1). Overall, 38% of retrieved articles were among the 100 top-ranked articles in the co-citation search.

### Factors affecting percentage of retrieved articles

Table 2 shows that the percentage of retrieved articles was higher when the query articles were cited more frequently and when their citations overlapped more (as indicated by the higher similarity score, see methods). The combined searches retrieved a median of 83% of the articles when all articles in the review were in PubMed versus 62% when fewer than 90% were in PubMed, suggesting that the method was less likely to retrieve articles that were obtained through other databases.

**Table 2:** Factors that influence the percentage of articles included in each review that were retrieved by co-citation and citation searches combined

	Number of reviews	Percentage of articles retrieved Median (IQR)
Number of citing articles		
< 20	7	37.5 (28.6, 60.0)
20–50	26	63.3 (39.4, 85.7)
50–100	48	65.0 (50.0, 87.5)
100–200	74	80.0 (50.0, 90.1)
> 200	95	77.6 (47.4, 93.8)
Similarity index*		
< 0.1	19	34.4 (15.4, 56.3)
0.1–0.2	26	56.8 (36.1, 72.3)
0.2–0.5	109	75.0 (51.7, 92.3)
> =0.5	96	83.3 (60.4, 94.4)
Percentage of articles in PubMed		
< 90	64	62.4 (41.3, 80.3)
90–100	64	67.1 (49.4, 84.5)
100	122	82.8 (59.6, 100.0)

*Similarity index = number of co-citations between query articles / number of citations of the less-cited query article. *IQR* inter-quartile range

Table 2 shows that in most reviews the query articles were cited more than 20 times and the similarity score between the query articles was greater than 0.2. Table 3 shows the percentage of retrieved articles for combinations of these criteria. When the number of citing articles was higher than 20, *and* the similarity score was higher than 0.2, CoCites retrieved a median of 80% of all included articles and 88% of included articles when all were in PubMed.

**Table 3:** Percentage of retrieved articles by the number of citing articles and similarity index

Number of citing articles	Similarity index*	Percentage of articles in PubMed	Number	Percentage retrieved, median
> 20	> 0.2	100	101	87.5 (68.0, 100.0)
> 100	> 0.2	100	70	90.8 (77.6, 100.0)
> 20	> 0.5	100	52	87.5 (77.4, 100.0)
> 100	> 0.5	100	32	91.9 (82.2, 100.0)
> 20	> 0.2	> 90	150	83.3 (64.1, 96.5)
> 100	> 0.2	> 90	111	85.7 (66.7, 96.7)
> 20	> 0.5	> 90	71	87.5 (69.2, 95.2)
> 100	> 0.5	> 90	47	87.5 (76.9, 95.2)
> 20	> 0.2	All	200	80.0 (60.0, 94.1)
> 100	> 0.2	All	139	81.2 (64.3, 95.2)
> 20	> 0.5	All	94	84.5 (61.5, 94.4)
> 100	> 0.5	All	59	86.8 (66.7, 94.4)

*Similarity index = number of co-citations between query articles / number of citations of the less-cited query article. *IQR* inter-quartile range

Even under these favorable conditions (Table 3, row 1), the method did not always work. We examined the citation characteristics of the twelve reviews in which CoCites retrieved fewer than 50% of the included articles (see Additional file 1: Table S1, Additional file 1). We observed that in 10 of 12 the reviews, co-citation searching retrieved 5 or fewer articles—too few to constitute an effective query set for the citation search. For 9 of these articles, citation searching retrieved no new publications. When CoCites failed to retrieve the articles that were included in the review, it nevertheless returned articles on the same topic as the query articles. Additional file 1: Table S2 (see Additional File 1) shows the titles of the top-ranked results for each of the five reviews in which the method performed worst, retrieving only 9–13% of the included articles. The titles show that most articles are on similar topics as the query articles.

In an exploratory analysis, we examined whether using the top-ranked results from the co-citation search could be effective as query articles for the *c*itation search. Based on the results of the citation searches, we opted to use the 25 top-ranked articles as query sets as this was shown to retrieve more than 50% of the included articles (see Additional file 1: Fig. S3, Additional File 1). We observed that the percentage of retrieved articles improved for all reviews. In 10 out of 12 reviews, more than 50% of included articles were retrieved, and in 6 of these, more than 75% were retrieved.

### Searching on the highest-cited article

For searches with high similarity scores (Table 3, row 1), we further examined performance of the method starting only with the highest-cited article. The co-citation search retrieved a median of 63.3% (IQR 45.3, 80.0) and the combined searches retrieved 86.7% (IQR 62.6, 100), results similar to those found when using the two highest-cited articles. (66.7 and 87.5%, respectively). Using only the highest-cited query article reduced the median number of screened articles from 873 (IQR 540, 1204) to 813 (IQR 394, 1165).

### Factors affecting retrieval of individual articles

Frequently cited articles were more likely to be retrieved by co-citation searching (Table 4); half of the infrequently-cited articles could be retrieved using citation searching. Articles that were not indexed in WOS were less likely to be retrieved. Articles that had never been cited could be retrieved using citation searching when they cited two or more articles that had already been retrieved by co-citation searching; in keeping with this finding, articles with more references were more likely to be retrieved. Finally, as expected, recently published articles were rarely retrieved because accumulating co-citations requires sufficient time since publication.

**Table 4:** Factors that affect retrieval of individual articles

		Co-citation search		Co-citation + citation searches	
	Total	Number retrieved	Percentage*	Number retrieved	Percentage*
Overall	4261	1938	45.5	2674	62.8
Times cited					
Not indexed	591	157	26.6	181	30.6
0	365	1	0.3	176	48.2
1–5	787	104	13.2	385	48.9
6–9	411	150	36.5	237	57.7
10–19	658	406	61.7	480	72.9
20–49	862	616	71.5	691	80.2
> 50	587	504	85.9	524	89.3
Indexed in WOS**					
No	703	184	26.2	235	33.4
Yes	3558	1754	49.3	2439	68.5
Number of references					
Not indexed	591	157	26.6	181	30.6
< 5	97	44	45.4	45	46.4
5–9	108	65	60.2	75	69.4
> =10	3465	1672	48.3	2373	68.5
Years since publication					
0–1	544	44	8.1	285	52.4
1–2	487	118	24.2	243	49..9
2–5	1081	402	37.2	606	56.1
5–10	994	585	58.9	686	69.0
> 10	1155	789	68.3	854	73.9

*Percentage of articles included in reviews that were retrieved, by category. Query articles for all 250 reviews (*n* = 500) were removed from the dataset. **All articles are in WOS but not all are indexed. See methods for details

### Finding the highly-cited articles

The new query articles were cited by a median of 25 articles (IQR 21, 35), which was markedly lower than the median of 160 when the two highest-cited articles were used. For all but one review, at least one of the two highest-cited articles was found among the 100 top-ranked results; in 68% of the reviews, both articles were in the top 100 (see Additional file 1: Table S3, Additional File 1). The ten top-ranked results included one (72%) or both (37%) of the highest-cited articles. In all but one review, the 100 top-ranked articles retrieved multiple other articles that were cited more frequently than the two query articles. In 34 reviews, the top 50 results retrieved 5 or more articles that were cited more frequently than the query articles.

## Discussion

In a well-defined, randomly selected sample of reviews, the combined use of CoCites’ co-citation and citation searches retrieved a median of 75% of the included articles. The method performed better when the query articles were more similar and more frequently cited. CoCites’ co-citation and citation searches combined retrieved 88% of included articles when all were in PubMed. In a subset of reviews with high similarity scores, the highest-cited articles could be retrieved when co-citation searching was based on less frequently cited articles.

Before discussing the implications of our findings, several methodological issues should be mentioned. First, we assumed that articles in the reviews were correctly included and excluded; however, it is possible that CoCites missed articles that should not have been included in the review and retrieved relevant articles that the authors had missed. If erroneous inclusion of articles is common, the performance of CoCites is underestimated.

And second, we only used the CoCites method while the authors of original reviews often utilized multiple sources, including foreign and specialty databases, conference proceedings, dissertations, and personal communications. The sources might yield articles that cannot be found through PubMed, WOS, or other major English-language literature databases. Therefore, it is not realistic to expect a 100% retrieval. When CoCites is used to find relevant articles for systematic reviews and meta-analyses, these additional sources may still need to be searched, if the topic so requires. There were other factors that may have under- or overestimated the performance too. We did not exclude articles that were not indexed in WOS even though they were less likely to be retrieved (Table 4), and, as Additional file 1: Table S2 (see Additional File 1) suggests, our study design may had limitations as well: the co-citation searches that performed worst did retrieve articles relevant for the topic of the review, just not the ones that were included in the review. On the contrary, the percentage of retrieved articles for each review was calculated including the two query articles, which overestimates the performance of the co-citation search. We kept the formulas as is because the query articles that were excluded from the co-citation search were often retrieved in the citation search.

We investigated the search method for the two highest-cited articles in each review, assuming that the researchers who performed the reviews were familiar with the topic and knew the two articles that most researchers knew and cited. Note that the query articles do not need to be the two highest-cited, they just need to be cited frequently enough. We showed in our pilot study that the combined searches worked equally well for various selections of the search articles [7], and showed here that the performance was similar when we only searched using one highest-cited article. To increase confidence in the performance of the co-citation search, the search can be repeated when it retrieves relevant articles that are cited more frequently than the query articles. These new query articles can be used to repeat the co-citation search and verify whether all findable relevant articles were retrieved.

In line with observations from our pilot study [7], we found that co-citation searching might not retrieve articles that are infrequently cited. These articles are more likely to include abstracts, letters, articles in non-English languages, and very old articles, reports, and theses that may not be indexed in WOS or other databases. Infrequently cited papers also include articles in WOS that were published too recently to be cited or that authors did not consider worth citing. Citation searching can retrieve such articles when they cite relevant articles in the query set. Further research is needed to assess the performance of the method in emerging and heterogeneous topics.

There is still room for improvement of the method. First, when query articles were on similar topics and cited frequently enough, CoCites retrieved fewer than 50% of the included articles in 12 reviews. The data in Additional file 1: Table S1 (see Additional file 1) showed that this was explained by the fact that the co-citation search did not retrieve enough articles for the citation search to be effective. When we applied the citation search to the 25 top-ranked co-citation search results instead (without screening relevant articles first), the percentage of retrieved articles increased substantially. These results warrant validation in a larger study, including further justification on how to determine the optimal number of query articles for a reliable citation search.

Second, for highly-cited query articles, we limited the number of titles needed to screen by requiring that titles in the results needed to be cited in 1% of the articles that cite the query articles. Yet, the data in Table 2 shows that the percentage of retrieved articles decreased when the query articles were cited more than 200 times, suggesting that the ‘1%’ threshold may not be optimal. An alternative and reproducible strategy is to only use the, say, 100 most recent articles that cite the query articles. As the CoCites method is inefficient for highly-cited query articles, it is worth exploring alternative strategies to limit the number needed to screen.

The performance of the CoCites method underscores that the degree of co-citation or co-citation frequency reflect the topic similarity between articles [8–11]: articles that are frequently co-cited are more likely on the same topic and thus articles that are (or belong) in the same review are more likely to be frequently co-cited. Similarly, ranking citations to and from multiple related query articles turns citation searching into a mix of co-citation searching and bibliographic coupling. Citation searching may not be efficient and effective when screening one query article at a time [4 5], but aggregating the results from multiple citation searches generates an informative ranking that again is based on the similarity of articles.

CoCites is useful for searches that aim to find related articles on a topic. Starting with one article, a CoCites search retrieves others that can be used to repeat the search or can be added to the query set. Although the method is relevant to any systematic review, it may be especially useful when the aim is to find related articles on a niche topic or identify the key, highest-cited publications. These key publications are easily retrieved among the top-ranked articles found by a co-citation search starting with query articles with fewer citations. Repeated iterations of the co-citation search, each time with the most highly cited relevant articles, will eventually reveal the highest-cited articles on the niche topic.

## Conclusions

CoCites is a novel method of searching scientific literature that retrieves related articles on well defined, specific topics. The method is effective and efficient and does not require expertise in building search queries. The method is transparent and reproducible. Co-citation searching has the potential to improve the quality and reduce the time of literature searches.

## Supplementary information

**Additional file 1: Figure S1.** Overview of the search methods. **Figure S2.** How CoCites works in practice: an example. **Figure S3.** Justification for selecting the 25 top-ranked articles. **Table S1.** Examples of reviews in which CoCites showed poor performance—and a possible solution. **Table S2.** Five examples of top-ranked results for the five reviews in which CoCites performed worst. **Table S3.** Finding highly cited articles using a search that starts with infrequently cited articles.

**Additional file 2.** Supplementary data.

## Data Availability

All data analyzed for this article are included in Additional file 2.

## Abbreviations

API: Application programming interface
IQR: Inter-quartile range
MOOSE: Meta-analysis Of Observational Studies in Epidemiology
PMID: PubMed identification number
PRISMA: Preferred Reporting Items for Systematic reviews and Meta-Analyses
WOS: Web of Science
