# Supplementary material for: Novel citation-based search method for scientific literature: a validation study
Source: BMC Med Res Methodol. 2020 Feb 7;20:25. doi: 10.1186/s12874-020-0907-5 (PMC7006380; doi:10.1186/s12874-020-0907-5)
Supplement: Supplementary file 1 — Additional file 1: Figure S1. Overview of the search methods. Figure S2. How CoCites works in practice: an example. Figure S3. Justification for selecting the 25 top-ranked articles. Table S1. Examples of reviews in which CoCites showed poor performance—and a possible solution. Table S2. Five examples of top-ranked results for the five reviews in which CoCites performed worst. Table S3. Finding highly cited articles using a search that starts with infrequently cited articles. [file 12874_2020_907_MOESM1_ESM.docx]

**Novel Citation-Based Search Method for Scientific Literature:**

**A Validation Study**

A.C.J.W. Janssens,*^1^ M. Gwinn^1^, J.E. Brockman^1^, K. Powell^2^, M. Goodman^1^

SUPPLEMENTARY TABLES AND FIGURES

**Supplementary Figure 1** Overview of the search methods

**Co-citation search**


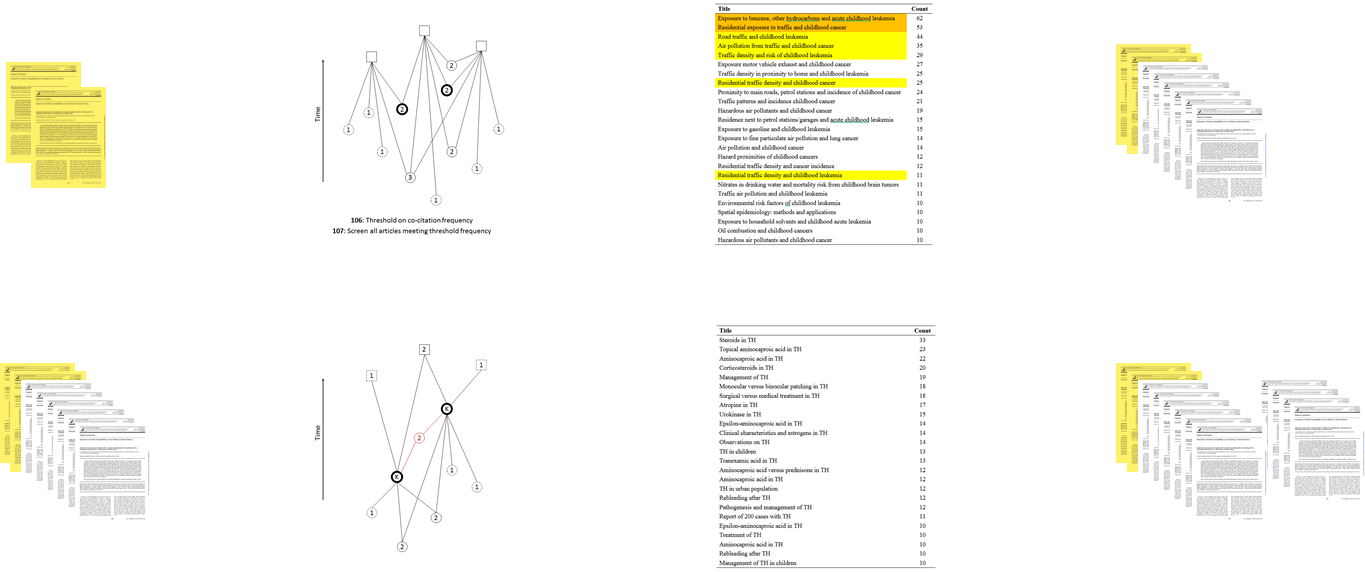
Query articles Search Screened articles Retrieved articles

**Citation search**

Query articles Search Screened articles Retrieved articles


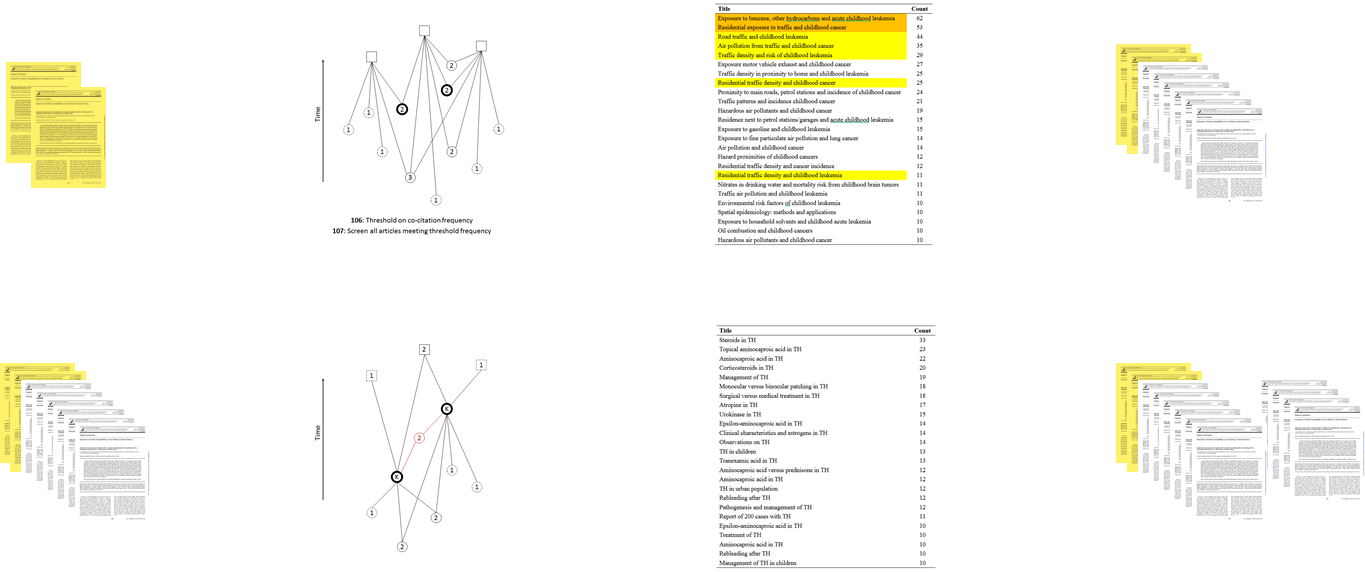


Articles retrieved by the co-citation search were used as query articles for the citation search. Searches were conducted using our web tool (see Methods). We screened results of both searches to find the articles included in the published review.

**Supplementary Figure 2** How CoCites works in practice: an example


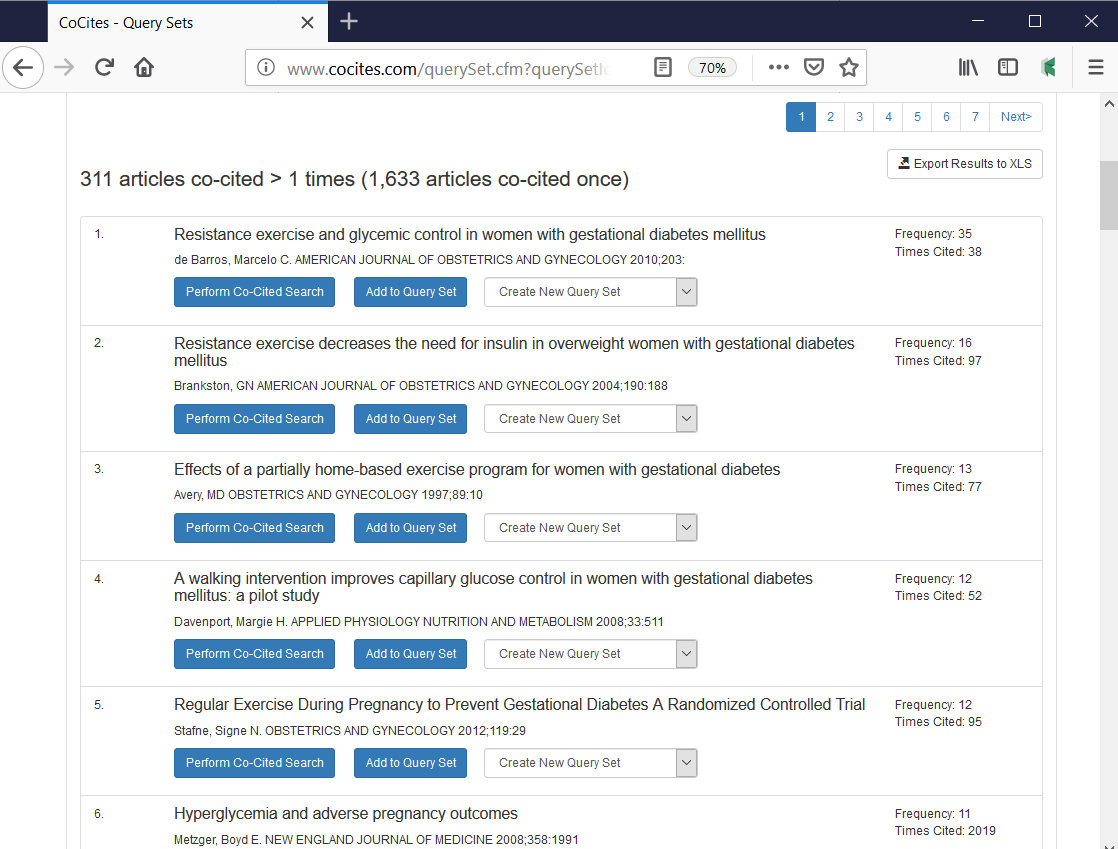

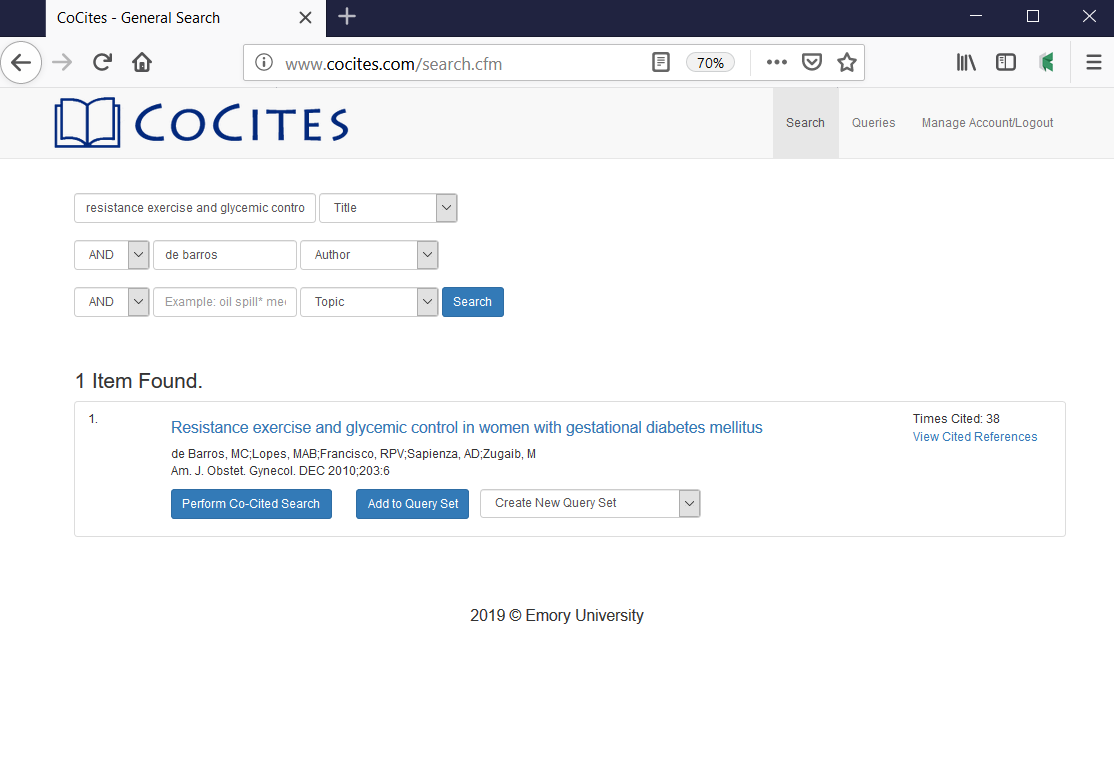


**Supplementary Figure 2** (continued)

The co-citation search returned 311 articles that were co-cited more than once with the article of De Barros *et al.* (Fig. 3b). The frequency is the number of times an article is co-cited with the query article. For example, the article of Brankston *et al.* is cited 97 times and co-cited with De Barros *et al.* 16 times.

The first article is the query article. Note that its frequency is 35 instead of 38, which means the article was cited 3 times by articles that are not indexed in WOS (see methods).

Fig. 3b

In this example, we aim to find related articles for:

De Barros *et al.* Resistance exercise and glycemic control in women with gestational diabetes mellitus. *Am J Obstet Gynecol* 2010.

The first step is to find this article in our web tool. Fig 3a shows that the article is cited 38 times.

To run the co-citation search, we click the button “Perform Co-Cited Search.”

Fig. 3a

|  | Title | Year | Times Cited | Freq |
| --- | --- | --- | --- | --- |
| 1 | Resistance **exercise** and glycemic control in women with **gestational diabetes** mellitus | 2010 | 38 | 35 |
| 2 | Resistance **exercise** decreases the need for insulin in overweight women with **gestational diabetes** mellitus | 2004 | 97 | 16 |
| 3 | Effects of a partially home-based **exercise** program for women with **gestational diabetes** | 1997 | 77 | 13 |
| 4 | A **walking** intervention improves capillary glucose control in women with **gestational diabetes** mellitus: a pilot study | 2008 | 52 | 12 |
| 5 | Regular **Exercise** During Pregnancy to Prevent **Gestational Diabetes** A Randomized Controlled Trial | 2012 | 95 | 12 |
| 6 | Hyperglycemia and adverse pregnancy outcomes | 2008 | 2019 | 11 |
| 7 | Randomized trial of diet versus diet plus **cardiovascular conditioning** on glucose-levels in **gestational diabetes** | 1989 | 126 | 9 |
| 8 | Prevention of **Gestational Diabetes** Feasibility issues for an **exercise** intervention in obese pregnant women | 2010 | 86 | 8 |
| 9 | A lifestyle intervention of weight-gain restriction: diet and **exercise** in obese women with **gestational diabetes** mellitus | 2007 | 119 | 8 |
| 10 | Simple **lifestyle** recommendations and the outcomes of **gestational diabetes**. A 2x2 factorial randomized trial | 2014 | 26 | 8 |
| 11 | No effect of the FitFor2 **exercise** programme on … in pregnant women who were overweight and at risk for **gestational diabetes** | 2012 | 73 | 7 |
| 12 | Guidelines of the American College of Obstetricians and Gynecologists for **exercise** during pregnancy and the postpartum period | 2003 | 299 | 7 |
| 13 | **Exercise** during **pregnancy** improves **maternal glucose** screen at 24-28 weeks: a randomised controlled trial | 2012 | 80 | 7 |
| 14 | **Physical Activity** Before and During Pregnancy and Risk of **Gestational Diabetes** Mellitus A meta-analysis | 2011 | 185 | 7 |
| 15 | A Multicenter, Randomized Trial of Treatment for Mild **Gestational Diabetes**. | 2009 | 931 | 7 |
| 16 | **Exercise** in **gestational diabetes** - an optional therapeutic approach | 1991 | 107 | 7 |
| 17 | Effect of treatment of **gestational diabetes** mellitus on pregnancy outcomes | 2005 | 1556 | 7 |
| 18 | Summary and recommendations of the Fifth International Workshop-Conference on **Gestational Diabetes** Mellitus | 2007 | 652 | 6 |
| 19 | A comparison of glyburide and insulin in women with **gestational diabetes** mellitus | 2000 | 474 | 6 |
| 20 | IAD-PGS Recommendations on the Diagnosis and Classification of **Hyperglycemia in Pregnancy** | 2010 | 1682 | 6 |
| 21 | Aerobic **exercise** and submaximal functional capacity in overweight pregnant women - A randomized trial | 2005 | 69 | 6 |
| 22 | Effect of **exercise** intensity and duration on ... glucose responses in pregnant women at low and high risk for **gestational diabetes** | 2012 | 31 | 6 |
| 23 | **Exercise** Training in **Pregnancy** Reduces Offspring Size without Changes in **Maternal Insulin** Sensitivity | 2010 | 123 | 6 |
| 24 | Home-Based **Exercise** Training Improves Capillary Glucose Profile in Women with **Gestational Diabetes** | 2014 | 25 | 6 |
| 25 | **Exercise** during pregnancy and **gestational diabetes**-related adverse effects: a randomised controlled trial | 2013 | 63 | 6 |

Fig. 3c

The table in Fig. 3c shows the top 25 results of the co-citation search. Like the query article, most articles are about exercise (red) and gestational diabetes (blue) and published around the same time as the query article or earlier. Note that the highly-cited articles (cited >500 times) in the list are ‘generic’ articles on gestational diabetes; all others are on exercise and gestational diabetes.

Next, to find recent related articles, we perform a citation search on the top 10 articles on exercise and gestational diabetes in Fig. 3c (which is articles #1-11, excluding #6). We select these articles and add them to the query set, see “Add to query set” button in Fig. 3b.

**Supplementary Figure 2** (continued)


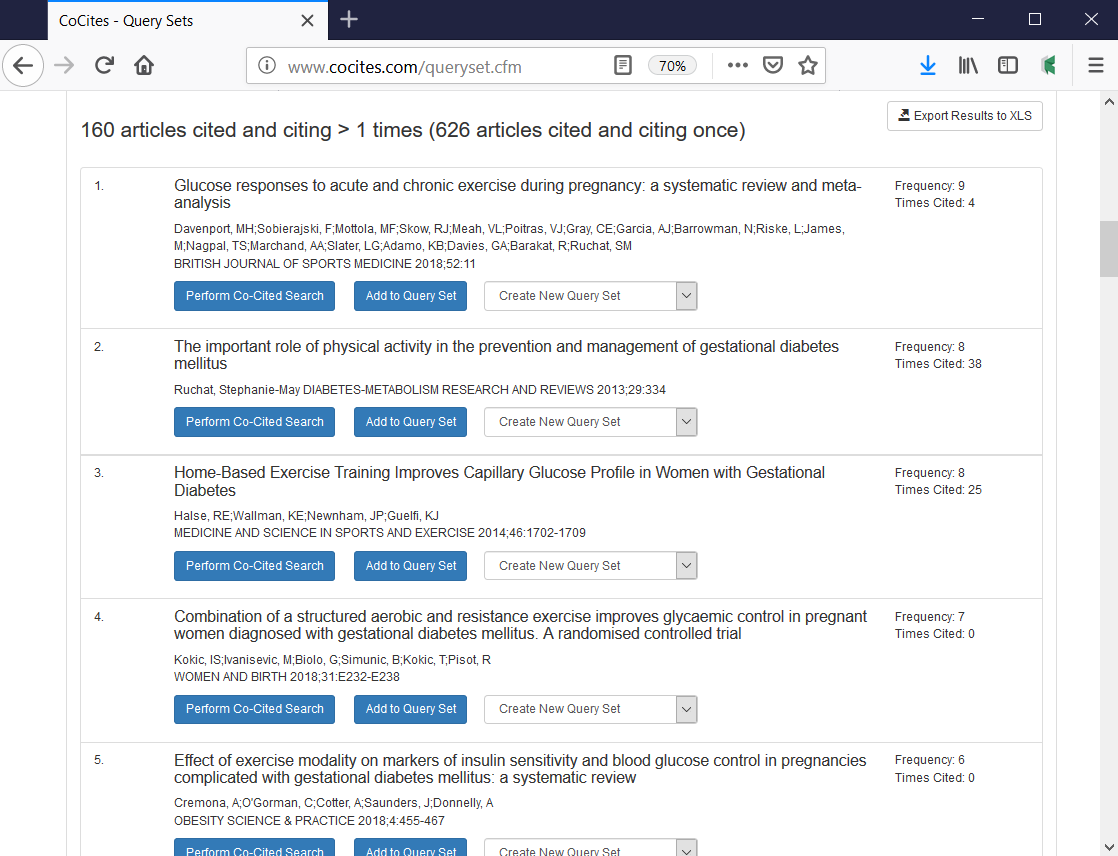
**Supplementary Figure 2** (continued)


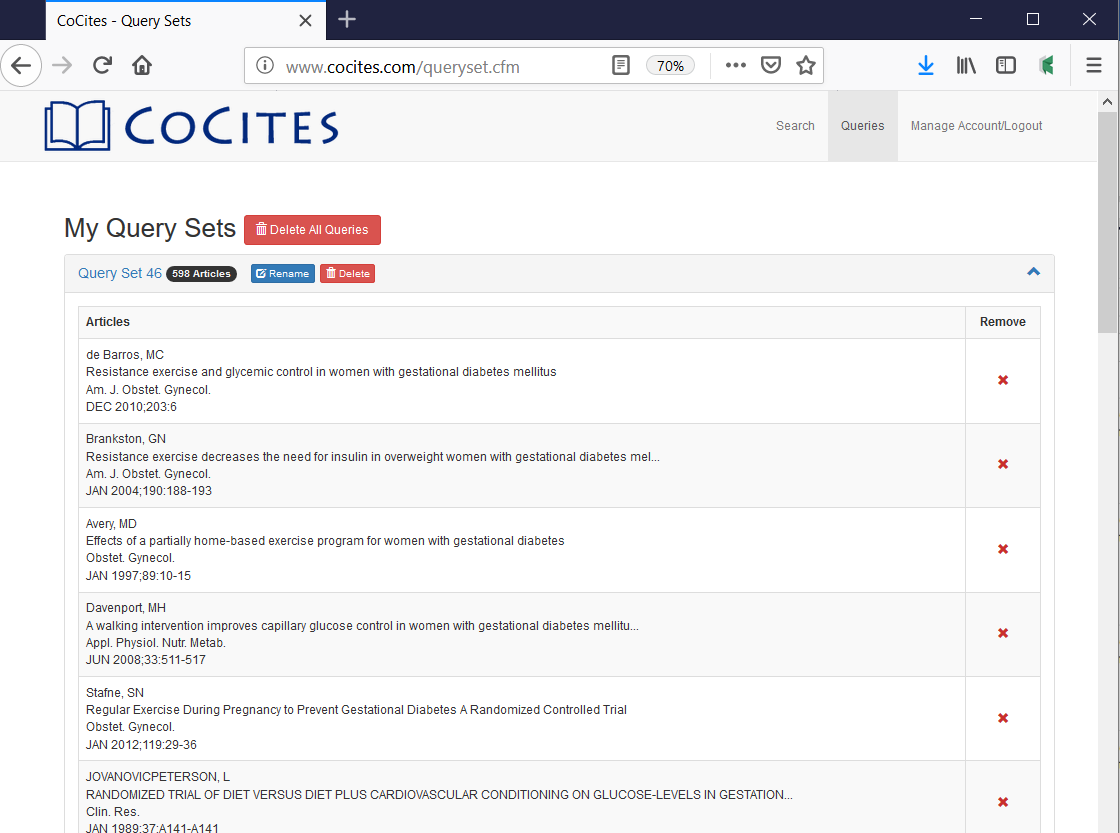

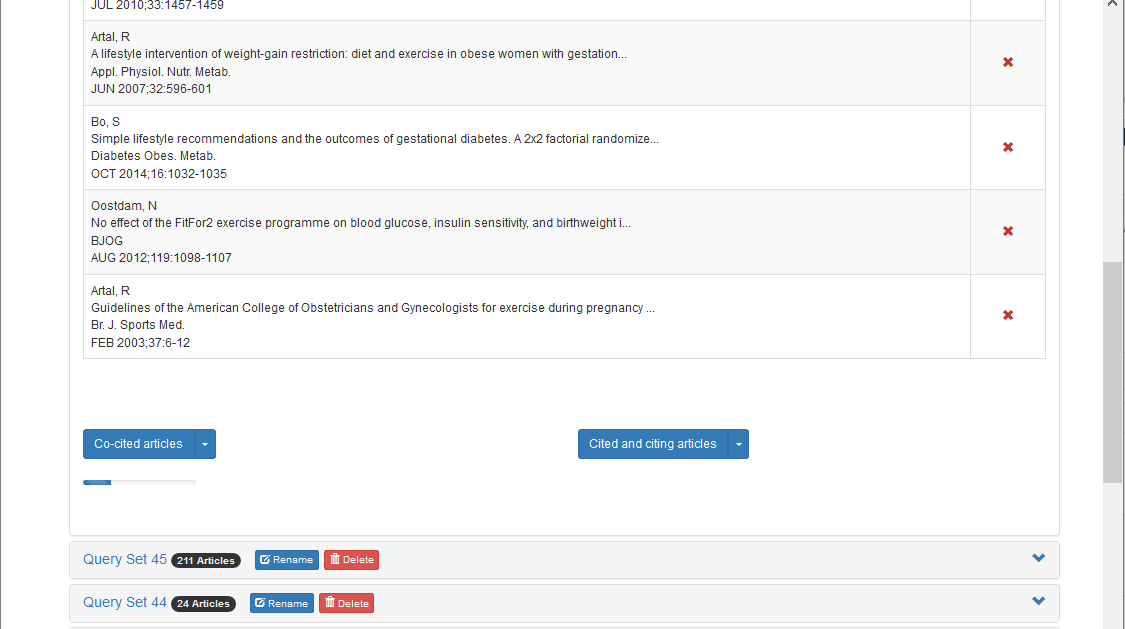

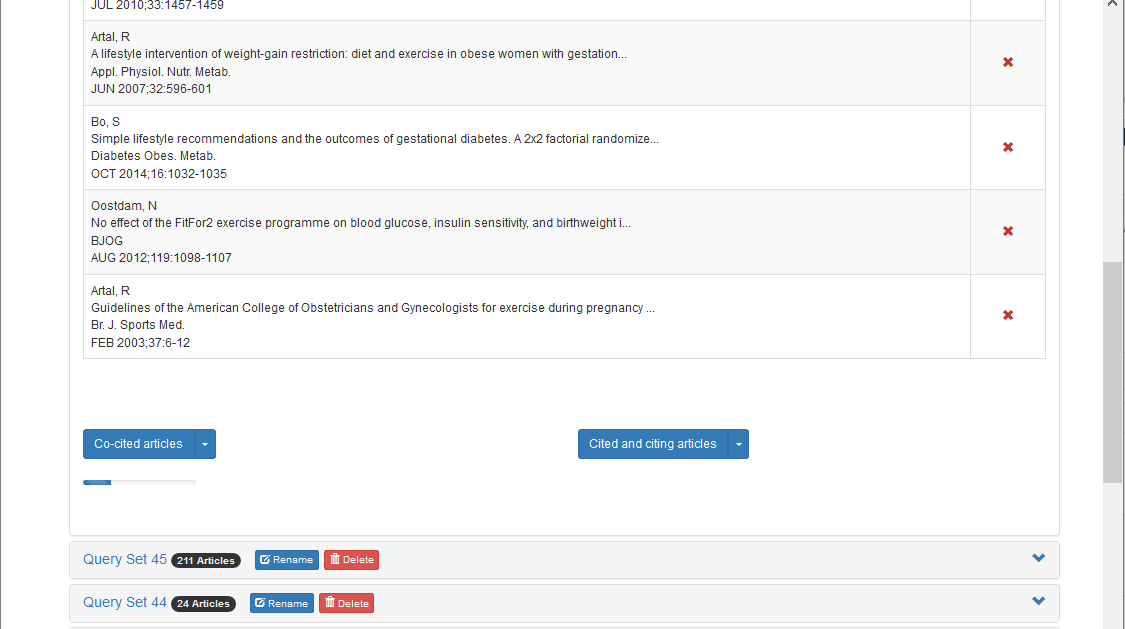


Fig. 3e shows the results of the citation search. The frequency is now the citation frequency. For each article, the frequency is the sum of the number of query articles that *cite* the article and the number of query articles that are *cited by* the article (see Methods).

Fig. 3e

Fig. 3d

Fig. 3d shows the new query set (the list is truncated presentation, the search included the 10 articles).

To run the citation search, we click the button “Cited and citing articles.” (The button “Co-cited articles” performs the same co-citation search as previous but on the entire query set.)

|  | Title | Year | Times Cited | Freq |
| --- | --- | --- | --- | --- |
| 1 | **Glucose** responses to acute and chronic **exercise** during **pregnancy**: a systematic review and meta-analysis | **2018** | 4 | 9 |
| 2 | The important role of **physical activity** in the prevention and management of **gestational diabetes** mellitus | 2013 | 38 | 8 |
| 3 | Home-Based **Exercise** Training Improves Capillary Glucose Profile in Women with **Gestational Diabetes** | 2014 | 25 | 8 |
| 4 | Combination of ... and resistance **exercise** improves glycaemic control in pregnant women ... with **gestational diabetes** mellitus | **2018** | 0 | 7 |
| 5 | Effect of **exercise** modality on … blood glucose control in pregnancies complicated with **gestational diabetes** mellitus | **2018** | 0 | 6 |
| 6 | Impact of prenatal **exercise** on neonatal and childhood outcomes: a systematic review and meta-analysis | **2018** | 4 | 6 |
| 7 | Simple **lifestyle** recommendations and the outcomes of **gestational diabetes**. A 2x2 factorial randomized trial | 2014 | 25 | 6 |
| 8 | **Exercise** and **gestational diabetes** mellitus | 2014 | 0 | 6 |
| 9 | **Physical Activity** and **Gestational Diabetes** Mellitus | 2014 | 5 | 6 |
| 10 | Resistance **Exercise** in Pregnancy and Outcome | 2016 | 2 | 5 |
| 11 | The efficacy of physiotherapy for the prevention and treatment of prenatal symptoms: a systematic review | 2015 | 1 | 5 |
| 12 | Randomized trial of diet versus diet plus **cardiovascular conditioning** on glucose-levels in **gestational diabetes** | 1989 | 126 | 5 |
| 13 | Interventions to improve **physical activity** during pregnancy: a systematic review on … | 2017 | 2 | 5 |
| 14 | The Effect of Self-care Educational/**Training** Interventions on the Outcomes of **Gestational Diabetes**: A Review Article | **2018** | 1 | 5 |
| 15 | **Physical activity** during pregnancy is associated with a lower prevalence of **gestational diabetes** mellitus in Vietnam | **2018** | 0 | 5 |
| 16 | Home-Based **Exercise** Improves Fitness and Exercise Attitude and Intention in Women with **GDM** | 2015 | 15 | 4 |
| 17 | **Exercise** improves glycaemic control in women diagnosed with **gestational diabetes** mellitus: a systematic review | 2016 | 9 | 4 |
| 18 | Effect of **physical activity** during pregnancy on mode of delivery | 2014 | 27 | 4 |
| 19 | Role of **exercise** in reducing the risks of **gestational diabetes** mellitus in obese women | 2016 | 0 | 4 |
| 20 | Treatments for women with **gestational diabetes** mellitus: an overview of Cochrane systematic reviews | **2018** | 1 | 4 |
| 21 | The Role of **Exercise** in the Prevention and Treatment of **Gestational Diabetes** Mellitus | 2008 | 22 | 4 |
| 22 | Resistance **exercise** decreases the need for insulin in overweight women with **gestational diabetes** mellitus | 2004 | 97 | 4 |
| 23 | Role of **Exercise** in Reducing **Gestational Diabetes** Mellitus | 2016 | 5 | 4 |
| 24 | **Exercise** for pregnant women with **gestational diabetes** for improving maternal and fetal outcomes | 2017 | 13 | 4 |
| 25 | **Exercise** Recommendations in Women with **Gestational Diabetes** Mellitus | 2010 | 3 | 4 |

Fig. 3f

The table in Fig. 3f shows the first 25 results of the citation search. Like the articles in the query set, all articles on the list are on exercise and gestational diabetes and most are of recent date.

The articles in the query set were published between 1997 and 2014, with one exception published in 1989. Thus, articles in the list that were published after 2014 are all *citing* the query articles, but article #12, published in 1989, must have been *cited by* 5 out of 10 query articles. The last article, published in 2010, was *citing* or *cited by* 4 query articles in total.

**Supplementary Figure 3** Justification for selecting the 25 top-ranked articles

**
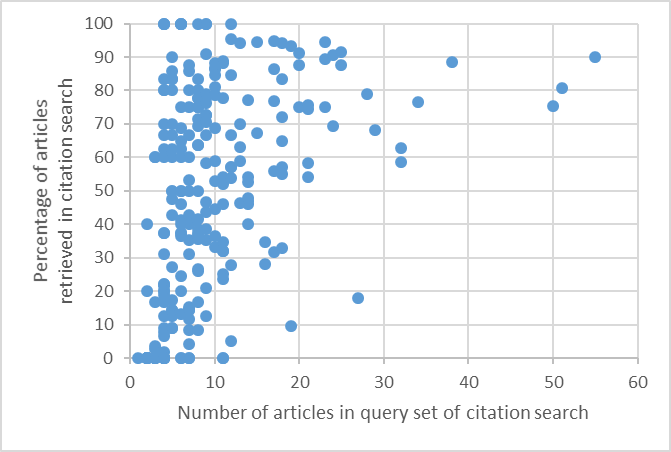
**

**Supplementary Table 1** Examples of reviews in which CoCites showed poor performance—and a possible solution

| \| Review \| Number included articles \| Number citing articles \| Similarity index \| Search 1: Co-citation ranking* \| Search 2: Citation ranking** \| Percentage retrieved articles \| Solution:  Citation Ranking Top 25 \| Percentage retrieved articles new \| \| --- \| --- \| --- \| --- \| --- \| --- \| --- \| --- \| --- \| \| 1 \| 10 \| 22 \| 0.27 \| 2 \| 0 \| 20% \| 1 \| 30% \| \| 2 \| 5 \| 293 \| 4.2*** \| 1 \| 0 \| 20% \| 3 \| 80% \| \| 3 \| 17 \| 260 \| 0.26 \| 3 \| 0 \| 27% \| 4 \| 64% \| \| 4 \| 7 \| 49 \| 0.36 \| 2 \| 0 \| 28% \| 5 \| 100% \| \| 5 \| 12 \| 141 \| 0.32 \| 3 \| 1 \| 33% \| 8 \| 92% \| \| 6 \| 14 \| 73 \| 0.31 \| 5 \| 0 \| 36% \| 1 \| 43% \| \| 7 \| 8 \| 58 \| 0.55 \| 3 \| 0 \| 38% \| 3 \| 75% \| \| 8 \| 16 \| 1779 \| 0.3 \| 4 \| 2 \| 38% \| 5 \| 56% \| \| 9 \| 13 \| 64 \| 0.46 \| 4 \| 1 \| 38% \| 3 \| 54% \| \| 10 \| 17 \| 82 \| 0.31 \| 7 \| 0 \| 41% \| 5 \| 71% \| \| 11 \| 25 \| 98 \| 0.73 \| 11 \| 0 \| 44% \| 8 \| 76% \| \| 12 \| 9 \| 486 \| 0.46 \| 4 \| 0 \| 44% \| 3 \| 78% \| |
| --- | --- | --- | --- | --- | --- | --- | --- | --- | --- | --- | --- | --- | --- | --- | --- | --- | --- | --- | --- | --- | --- | --- | --- | --- | --- | --- | --- | --- | --- | --- | --- | --- | --- | --- | --- | --- | --- | --- | --- | --- | --- | --- | --- | --- | --- | --- | --- | --- | --- | --- | --- | --- | --- | --- | --- | --- | --- | --- | --- | --- | --- | --- | --- | --- | --- | --- | --- | --- | --- | --- | --- | --- | --- | --- | --- | --- | --- | --- | --- | --- | --- | --- | --- | --- | --- | --- | --- | --- | --- | --- | --- | --- | --- | --- | --- | --- | --- | --- | --- | --- | --- | --- | --- | --- | --- | --- | --- | --- | --- | --- | --- | --- | --- | --- | --- | --- | --- |

These twelve reviews had all included articles in PubMed, a similarity index higher than 0.2, and more than 20 citing articles (Table 3, first row). The percentage of retrieved articles refers to the results of both searches combined. The proposed solution applied citation ranking to the 25 top-ranked results of co-citation ranking (see Methods).

* Includes the two query articles.

** Includes only articles retrieved in addition to those found by co-citation ranking.

*** This is one of the two reviews for which the similarity index was out of bounds (>1). In this case, the query articles were cited 427 and 5 times. The total number of citations is then 432, but the number of unique citations was 399. The difference between these numbers indicates the overlap in citations but is here larger than the lowest number of citations. This is possible because we can only obtain unique citations to articles that are indexed in WOS, whereas the citation count includes non-indexed articles too.

**Supplementary Table 2** Five examples of top-ranked results for the five reviews in which C performed worst.

Red numbers indicate query articles

| Beyond the mean: A systematic review on the correlates of daily intraindividual variability of **sleep/wake patterns** | | | |
| --- | --- | --- | --- |
| Bei et al. SLEEP MEDICINE REVIEWS. 2016. | | | |
| **Percentage of included articles retrieved: 9%** | | | |
|  |  |  |  |
|  | Title | Times Cited | Frequency |
| **1** | The **sleep** of remitted bipolar outpatients: a controlled naturalistic study using actigraphy | 112 | 59 |
| **2** | Daily activities and **sleep** quality in college students | 107 | 58 |
| 3 | **Sleep**-related functioning in euthymic patients with bipolar disorder, patients with insomnia, … | 196 | 39 |
| 4 | Actigraphic assessment of **circadian activity** and **sleep patterns** in bipolar disorder | 163 | 36 |
| 5 | THE PITTSBURGH **SLEEP** QUALITY INDEX - A NEW INSTRUMENT FOR PSYCHIATRIC PRACTICE AND RESEARCH | 8290 | 29 |
| 6 | A systematic review of manic and depressive prodromes | 198 | 24 |
| 7 | **Sleep** and **circadian rhythms** in bipolar disorder: Seeking synchrony, harmony, and regulation | 264 | 24 |
| 8 | Temporal relation between **sleep** and mood in patients with bipolar disorder | 106 | 19 |
| 9 | Social zeitgebers and **biological rhythms** – a unified approach to understanding the etiology of depression | 346 | 18 |
| 10 | A self assessment questionnaire to determine morningness eveningness in human **circadian rhythms** | 2302 | 17 |
| 11 | **Sleep schedules** and daytime functioning in adolescents | 867 | 16 |
| 12 | Diagnostic and Statistical Manual of Mental Disorders: DSM-IV TR | 42341 | 16 |
| 13 | RATING-SCALE FOR MANIA - RELIABILITY, VALIDITY AND SENSITIVITY | 4434 | 16 |
| 14 | Rate of switch from depression into mania after therapeutic **sleep** deprivation in bipolar depression | 164 | 16 |
| 15 | Interpersonal and social rhythm therapy: Managing the chaos of bipolar disorder | 301 | 15 |
| 16 | **SLEEP REDUCTION** AS A FINAL COMMON PATHWAY IN THE GENESIS OF MANIA | 255 | 15 |
| 17 | Morning-evening preference: **Sleep pattern spectrum** and lifestyle habits among Japanese junior high school pupils | 92 | 15 |
| 18 | **Circadian activity rhythm abnormalities** in ill and recovered bipolar I disorder patients | 85 | 15 |
| 19 | THE SOCIAL RHYTHM METRIC - AN INSTRUMENT TO QUANTIFY THE **DAILY RHYTHMS** OF LIFE | 159 | 13 |
| 20 | Evaluation of 3 **circadian-rhythm** questionnaires with suggestions for an improved measure of morningness | 521 | 13 |
| 21 | The prospective impact of **sleep** duration on depression and mania | 68 | 13 |
| 22 | The relationship between lifestyle regularity and subjective **sleep** quality | 55 | 12 |
| 23 | Two-year outcomes for interpersonal and social rhythm therapy in individuals with bipolar I disorder | 390 | 12 |
| 24 | Recommendations for a standard research assessment of **insomnia** | 487 | 12 |
| 25 | **Circadian** preference, **sleep** and daytime behaviour in adolescence | 357 | 12 |

| **Taxane acute pain syndrome (TAPS)** in patients receiving **taxane-based chemotherapy** for **breast cancer**-a systematic review | | | |
| --- | --- | --- | --- |
| Fernandes et al. SUPPORTIVE CARE IN CANCER. 2016. | | | |
| **Percentage of included articles retrieved: 10%** | | | |
|  |  |  |  |
|  | Title | Times Cited | Frequency |
| **1** | Phase III trial comparing … with **docetaxel** plus cyclophosphamide as adjuvant therapy for operable **breast cancer** | 279 | 228 |
| **2** | Doxorubicin and **paclitaxel** versus … as first-line therapy for women with metastatic **breast cancer**: … trial | 210 | 198 |
| 3 | Adjuvant **docetaxel** for node-positive **breast cancer** | 625 | 115 |
| 4 | Effects of **chemotherapy** and hormonal therapy for early **breast cancer** on recurrence and 15-year survival: … | 3754 | 104 |
| 5 | Improved outcomes from adding sequential **paclitaxel** but not from escalating doxorubicin dose in an adjuvant **chemotherapy** regimen for patients with node-positive primary **breast cancer** | 736 | 101 |
| 6 | Use of **chemotherapy** plus a monoclonal antibody against HER2 for metastatic **breast cancer** that overexpresses HER2. | 6133 | 97 |
| 7 | **Docetaxel** and … as first-line **chemotherapy** for metastatic **breast cancer**: Results of a … phase III trial | 244 | 95 |
| 8 | Doxorubicin and **paclitaxel** versus … as first-line **chemotherapy** in metastatic **breast cancer**: The EORTCancer … trial | 152 | 94 |
| 9 | Phase III trial of doxorubicin, **paclitaxel**, and the combination … as … **chemotherapy** for metastatic **breast cancer**: … | 400 | 91 |
| 10 | Sequential adjuvant epirubicin-based and **docetaxel chemotherapy** for node-positive **breast cancer** patients: … | 364 | 89 |
| 11 | Superior survival with capecitabine plus **docetaxel** combination **therapy** in … advanced **breast cancer**: … | 723 | 86 |
| 12 | Prospective randomized trial of **docetaxel** versus doxorubicin in patients with metastatic **breast cancer** | 489 | 84 |
| 13 | Randomized trial of … **chemotherapy** as postoperative adjuvant treatment of node-positive primary **breast cancer**: … | 874 | 81 |
| 14 | Trastuzumab plus adjuvant **chemotherapy** for operable HER2-positive **breast cancer** | 3088 | 76 |
| 15 | **Paclitaxel** after doxorubicin plus cyclophosphamide as adjuvant **chemotherapy** for node-positive **breast cancer**: … | 360 | 68 |
| 16 | Trastuzumab after adjuvant **chemotherapy** in HER2-positive **breast cancer** | 2785 | 66 |
| 17 | **Docetaxel** With Cyclophosphamide Is Associated With an Overall Survival Benefit Compared With Doxorubicin and …. | 260 | 64 |
| 18 | Prospective randomized trial of **docetaxel** versus mitomycin plus vinblastine in patients with metastatic **breast cancer** … | 408 | 62 |
| 19 | **Paclitaxel** versus doxorubicin as first-line single-agent **chemotherapy** for metastatic **breast cancer**: … | 245 | 59 |
| 20 | Weekly **paclitaxel** in the adjuvant treatment of **breast cancer** | 422 | 49 |
| 21 | Cytotoxic and hormonal treatment for metastatic **breast cancer**: A systematic review of published randomized trials … | 360 | 48 |
| 22 | Randomized phase III study of **docetaxel** compared with **paclitaxel** in metastatic **breast cancer** | 305 | 47 |
| 23 | Initial **paclitaxel** improves outcome compared with … **chemotherapy** as front-line therapy in … **breast cancer** | 144 | 47 |
| 24 | **Paclitaxel** by 3-hour infusion in combination with … in women with untreated metastatic **breast cancer** … | 494 | 43 |
| 25 | **Paclitaxel** plus bevacizumab versus **paclitaxel** alone for metastatic **breast cancer** | 1890 | 41 |

| Association between **maternal nutritional** status in **pregnancy** and **offspring cognitive function** during childhood and adolescence; a systematic review | | | |
| --- | --- | --- | --- |
| Veena et al. BMC PREGNANCY AND CHILDBIRTH. 2016. | | | |
| **Percentage of included articles retrieved: 11%** | | | |
|  |  |  |  |
|  | Title | Times Cited | Frequency |
| **1** | **Maternal vitamin D** status during **pregnancy** and **child outcomes** | 322 | 216 |
| 2 | **Maternal vitamin D** intake during **pregnancy** and early **childhood** wheezing | 356 | 107 |
| 3 | **Maternal intake** of **vitamin D** during **pregnancy** and risk of recurrent wheeze in **children** at 3 y of age | 391 | 105 |
| 4 | Infant vitamin D supplementation and allergic conditions in adulthood - Northern Finland Birth Cohort 1966 | 224 | 90 |
| 5 | **Maternal vitamin D intake** during **pregnancy** is inversely associated with asthma and … in 5-year-old **children** | 236 | 82 |
| 6 | Vitamin D deficiency | 5721 | 74 |
| **7** | **Neurodevelopmental effects** of **maternal nutritional** status and … **methylmercury** from … **fish** during **pregnancy** | 99 | 72 |
| 8 | **Cord-Blood** **25-Hydroxyvitamin D** Levels and Risk of Respiratory Infection, Wheezing, and Asthma | 234 | 57 |
| 9 | Dairy **food, calcium** and **vitamin D intake** in **pregnancy**, and wheeze and eczema in **infants** | 146 | 53 |
| 10 | Is vitamin D deficiency to blame for the asthma epidemic? | 272 | 52 |
| 11 | Serum Vitamin D Levels and Markers of Severity of **Childhood** Asthma in Costa Rica | 317 | 50 |
| 12 | **Maternal vitamin D** status during **pregnancy** and **childhood** bone mass at age 9 years: a longitudinal study | 405 | 46 |
| 13 | Reversing the defective induction of IL-10-secreting regulatory T cells in glucocorticoid-resistant asthma patients | 353 | 40 |
| 14 | Randomized trial of vitamin D supplementation to prevent seasonal influenza A in school**children** | 351 | 38 |
| 15 | Relationship between serum 25-hydroxyvitamin D and pulmonary function in the Third NHANES | 322 | 37 |
| 16 | **Maternal 25-hydroxyvitamin D** and parathyroid hormone concentrations and **offspring** birth size | 172 | 37 |
| 17 | **Cognitive** deficit in 7-year-old **children** with **prenata**l exposure to **methylmercury** | 970 | 36 |
| 18 | Toll-like receptor triggering of a vitamin D-mediated human antimicrobial response | 1831 | 35 |
| 19 | Serum 25-hydroxyvitamin D and IgE - a significant but nonlinear relationship | 119 | 35 |
| 20 | **Maternal vitamin D** deficiency increases the risk of preeclampsia | 363 | 34 |
| 21 | **VITAMIN-D** SUPPLEMENTS IN **PREGNANT** ASIAN WOMEN - EFFECTS ON CALCIUM STATUS AND **FETAL GROWTH** | 236 | 33 |
| 22 | Genes, factor X, and allergens: what causes allergic diseases? | 80 | 32 |
| 23 | Does **Vitamin D Intake** During **Infancy** Promote the Development of Atopic Allergy? | 100 | 32 |
| 24 | **Vitamin D** Supplementation During **Pregnancy**: Double-Blind, Randomized Clinical Trial of Safety and Effectiveness | 292 | 31 |
| 25 | **Prenatal methylmercury** exposure from ocean **fish consumption** in the Seychelles **child development** study | 327 | 31 |

| A Systematic Review of **Metastatic Hepatocellular Carcinoma** to the **Spine** | | | |
| --- | --- | --- | --- |
| Goodwin et al. WORLD NEUROSURGERY. 2016. | | | |
| **Percentage of included articles retrieved: 12%** | | | |
|  |  |  |  |
|  | Title | Times Cited | Frequency |
| **1** | Advanced **hepatocellular carcinoma** with distant **metastases**, successfully treated by a combination therapy of … | 38 | 36 |
| **2** | Percutaneous **sacroplasty** for hemorrhagic **metastases** from **hepatocellular carcinoma** | 34 | 26 |
| 3 | Combined … therapy for **advanced** **hepatocellular carcinoma** with **tumor** thrombi in the major portal branches | 164 | 24 |
| 4 | Augmentation of … is associated with up-regulation of p27(Kip1) in human **hepatocellular carcinoma** cells | 75 | 15 |
| 5 | Clinical pilot study of <therapy> for patients with locally **advanced hepatocellular carcinoma** | 79 | 14 |
| 6 | Treatment of **hepatocellular carcinoma** with <therapy>; role of type I interferon receptor expression | 104 | 14 |
| 7 | Percutaneous sacroplasty for the treatment of **sacral insufficiency fractures** | 64 | 14 |
| 8 | **Sacroplasty** by CT and fluoroscopic guidance: Is the procedure right for your patient? | 35 | 13 |
| 9 | Antineoplastic activity of the combination of <therapy> against experimental and human **malignancies** | 355 | 13 |
| 10 | Combination therapy … for **advanced hepatocellular carcinoma** with portal venous invasion | 151 | 13 |
| 11 | Partial contribution of … pathway to antitumor effects of <therapy> against **hepatocellular carcinoma** | 61 | 12 |
| 12 | Interferon alfa receptor expression and growth inhibition by interferon alfa in human **liver cancer** cell lines | 116 | 12 |
| 13 | Percutaneous **sacroplasty**: Long-axis injection technique | 36 | 12 |
| 14 | Percutaneous osteoplasty as a treatment for painful malignant bone lesions of the pelvis and femur | 59 | 12 |
| 15 | **Sacroplasty**: A treatment for **sacral insufficiency fractures** | 80 | 12 |
| 16 | **Sacroplasty**: A new treatment for **sacral insufficiency fracture** | 88 | 11 |
| 17 | Percutaneous **sacroplasty** for osteoporotic **sacral insufficiency fractures**: a … pilot study | 73 | 11 |
| 18 | PMMA cementoplasty in symptomatic **metastatic lesions** of the S1 **vertebral** body | 39 | 11 |
| 19 | CT-guided **sacroplasty** in advanced **sacral destruction** secondary to **tumour** infiltration | 15 | 11 |
| 20 | Integration of interferon-alpha/beta signalling to p53 responses in **tumour** suppression and antiviral defence | 542 | 11 |
| 21 | <therapy> induces apoptosis through IFN-alpha/beta receptor in human **hepatocellular carcinoma** cells | 57 | 11 |
| 22 | TOXICITY AND RESPONSE CRITERIA OF THE EASTERN-COOPERATIVE-ONCOLOGY-GROUP | 4743 | 11 |
| 23 | Randomized controlled trial of interferon treatment for **advanced hepatocellular carcinoma** | 175 | 10 |
| 24 | A novel chemotherapy for **advanced hepatocellular carcinoma** with tumor thrombosis of the main trunk … | 123 | 10 |
| 25 | Phase II trial of <therapy> for treatment of **hepatocellular carcinoma** | 112 | 10 |

| Impact of **lifestyle interventions** on **depressive symptoms** in individuals at-risk of, or with, type 2 **diabetes** mellitus: A systematic review and meta-analysis of randomized controlled trials | | | |
| --- | --- | --- | --- |
| Cezaretto et al. NUTRITION METABOLISM AND CARDIOVASCULAR DISEASES. 2016. | | | |
| **Percentage of included articles retrieved: 13%** | | | |
|  |  |  |  |
|  | Title | Times Cited | Frequency |
| **1** | The pathways study - A randomized trial of **collaborative care** in patients with **diabetes** and **depression** | 407 | 333 |
| **2** | Effectiveness of the **diabetes education** and **self management** for ongoing and newly diagnosed (DESMOND) programme for people with newly diagnosed **type 2 diabetes**: cluster randomised controlled trial | 283 | 183 |
| 3 | The prevalence of comorbid **depression** in adults with **diabetes** - A meta-analysis | 1767 | 113 |
| 4 | **Collaborative** **care management** of late-life **depression** in the primary care setting - A randomized controlled trial | 1242 | 98 |
| 5 | Association of **depression** and **diabetes** complications: A meta-analysis | 804 | 86 |
| 6 | Cognitive **behavior therapy** for **depression** in **type 2 diabetes** mellitus - A randomized, controlled trial | 325 | 82 |
| 7 | The effectiveness of **depression** **care management** on **diabetes**-related outcomes in older patients | 178 | 82 |
| 8 | **Depression** and **poor glycemic control** - A meta-analytic review of the literature | 849 | 77 |
| 9 | **Depression** and **diabetes** - Impact of **depression** symptoms on adherence, function, costs | 791 | 73 |
| 10 | The PHQ-9 - Validity of a brief **depression** severity measure | 6255 | 67 |
| 11 | Fluoxetine for **depression** in **diabetes** - A randomized double-blind placebo-controlled trial | 271 | 62 |
| 12 | Effects of nortriptyline on **depression** and glycemic control in **diabetes**: Results of a … trial | 254 | 60 |
| 13 | **Collaborative Care** for Patients with **Depression** and Chronic Illnesses. | 589 | 53 |
| 14 | The association of comorbid **depression** with mortality in patients with **type 2 diabetes** | 317 | 50 |
| 15 | **Collaborative care** for **depression** - A cumulative meta-analysis and review of longer-term outcomes | 636 | 49 |
| 16 | Relationship of **depression** and **diabetes** **self-care**, medication adherence, and preventive care | 452 | 49 |
| 17 | **Depression** predicts increased incidence of adverse health outcomes in older Mexican Americans with **type 2 diabetes** | 287 | 47 |
| 18 | **Collaborative management** to achieve treatment guidelines – impact on **depression** in primary care | 1008 | 47 |
| 19 | Cost-effectiveness of systematic **depression** treatment among people with **diabetes mellitus** | 171 | 46 |
| 20 | Comorbid **depression** is associated with increased health care use and expenditures in individuals with **diabetes** | 376 | 45 |
| 21 | Validation and utility of a self-report version of PRIME-MD - The PHQ primary care study | 3470 | 42 |
| 22 | Structured patient **education**: the **Diabetes** X-PERT Programme makes a difference | 150 | 41 |
| 23 | Effectiveness of **self management training** in **type 2 diabetes** - A systematic review of randomized controlled trials | 955 | 39 |
| 24 | The prevalence of co-morbid **depression** in adults with **Type 2 diabetes**: a systematic review and meta-analysis | 466 | 37 |
| 25 | **Self-management education** for adults with **type 2 diabetes** - A meta-analysis of the effect on **glycemic control** | 739 | 36 |

**Supplementary Table 3** Finding highly cited articles using a search that starts with infrequently cited articles

| a. Number of reviews for which (second) highest-cited query articles were retrieved at ranking threshold | | | | | | | |
| --- | --- | --- | --- | --- | --- | --- | --- |
|  | Ranking threshold | | | | All | Not found | Total |
|  | Top 10 | Top 25 | Top 50 | Top 100 |  |  |  |
| Highest-cited | 43 | 50 | 53 | 59 | 59 | 1 | 60 |
| Second highest-cited | 22 | 33 | 38 | 41 | 54 | 6 | 60 |
| Total | 65 | 83 | 91 | 100 | 113 | 7 | 120 |

| b. Number of included articles that were retrieved at ranking threshold* | | | | | |
| --- | --- | --- | --- | --- | --- |
|  | Ranking threshold | | | |  |
|  | Top 10 | Top 25 | Top 50 | Top 100 | All |
| None | 8 | 3 | 3 | 1 | 1 |
| 1-4 | 48 | 31 | 23 | 19 | 17 |
| 5-9 | 4 | 26 | 30 | 32 | 30 |
| >=10 | 0 | 0 | 4 | 8 | 12 |
| Total | 60 | 60 | 60 | 60 | 60 |

This analysis was restricted to the reviews (n=60) that had all included articles in PubMed, a similarity index higher than 0.2, and more than 20 citing articles (Table 3, row 1) and had included 10 or more articles.

* The two highest-cited query articles are not considered.
